# Effects and Mechanisms of Transcutaneous Electroacupuncture on Chemotherapy-Induced Nausea and Vomiting

**DOI:** 10.1155/2014/860631

**Published:** 2014-08-31

**Authors:** Xing Zhang, Hai-feng Jin, Yi-hong Fan, Bin LU, Li-na Meng, Jiande D. Z. Chen

**Affiliations:** ^1^Division of Gastroenterology, The First Affiliated Hospital of Zhejiang Chinese Medical University, Hangzhou 310006, China; ^2^Division of Gastroenterology, Sixth People's Hospital of Shaoxing, Shaoxing 312000, China; ^3^Ningbo Pace Translational Medical Research Center, Beilun, Ningbo 315043, China; ^4^Division of Gastroenterology and Hepatology, Johns Hopkins University, Baltimore, MD 21224, USA

## Abstract

Nausea and vomiting are one of the major complications of chemotherapy for cancers. The aim of this study is to investigate the emetic effects and mechanisms involving serotonin and dopamine of needleless transcutaneous electroacupuncture (TEA) at Neiguan (PC6) and Jianshi (PC5) on chemotherapy-induced nausea and vomiting in patients with cancers. Seventy-two patients with chemotherapy were randomly divided into sham-TEA group (sham-TEA, *n* = 34) and TEA group (*n* = 38). TEA was performed at PC 6 and PC 5 (1 h, bid) in combination with granisetron. Sham-TEA was delivered at nonacupoints using the same parameters. We found the following. (1) In the acute phase, the conventional antiemetic therapy using Ondansetron effectively reduced nausea and vomiting; the addition of TEA did not show any additive effects. In the delayed phase, however, TEA significantly increased the rate of complete control (*P* < 0.01) and reduced the nausea score (*P* < 0.05), compared with sham-TEA. (2) TEA significantly reduced serum levels of 5-HT and dopamine in comparison with sham-TEA. Those results demonstrate that needleless transcutaneous electroacupuncture at PC6 using a watch-size digital stimulator improves emesis and reduces nausea in the delayed phase of chemotherapy in patients with cancers. This antiemetic effect is possibly mediated via mechanisms involving serotonin and dopamine.

## 1. Introduction

Chemotherapy is an important component of comprehensive treatments for cancers. Nausea and vomiting are one of the major complications of chemotherapy. Chemotherapy-induced nausea and vomiting (CINV) lead to a variety of adverse clinical consequences, including noncompliance with therapy, undermining of the efficacy of therapy, and unwillingness or even refusal of therapy [1–3].

Antiemetics include 5-HT_3_ receptor antagonists, glucocorticoids, dopamine receptor antagonists, benzodiazepine class of drugs, antipsychotic drugs, and marijuana. Among them, 5-HT_3_ receptor antagonists are most widely used [4]. Introduction of 5-HT_3_ receptor antagonists in the early 1990s represents major advance in the management of acute CINV. Common adverse events of 5-HT_3_ receptor antagonists include mild headache, transient increase in hepatic transaminase level, and constipation [5]. The major problems with the 5-HT_3_ receptor antagonist are (1) lack of efficacy in treating delayed emesis and (2) lack of efficacy in treating nausea in both acute and delayed phases [6]. According to the functional living index, nausea was reported to have a stronger negative impact on patients' daily life than vomiting [7]. Neither clinical evidence nor the ratio of cost/effectiveness justifies the use of the 5-HT_3_ antagonist beyond 24 hours after chemotherapy for prevention of delayed emesis. Therefore, the outcome of the treatment for CINV is unsatisfactory and there is still an urgent need for the development of novel therapies for CINV, especially delayed CINV.

Acupuncture has been used to treat nausea and vomiting in China for thousands of years. The most commonly used acupoints for the treatment of gastrointestinal symptoms are Neiguan (PC6), Zusanli (ST36), and Jianshi (PC5). A large number of studies have demonstrated that acupuncture or electroacupuncture (EA) can effectively reduce nausea and vomiting under various conditions, such as postsurgery [8–10], pregnancy [11, 12], and motion sickness [13]. Dundee et al. reported that acupuncture treatment might also significantly reduce CINV [14, 15]. Acupuncture and EA are performed by acupuncturists or doctors due to the insertion of needles into the acupoints and therefore the patient can receive the treatment only in clinics or hospitals. To make the therapy readily available at patient's home, a needleless self-administrated method of transcutaneous electroacupuncture (TEA) was proposed in this study.

The aim of this study was to investigate the emetic effects and mechanisms involving serotonin and dopamine of the proposed needleless TEA at PC6 and PC5 on CINV in patients with cancers.

## 2. Material and Methods

### 2.1. Study Population

The study was conducted according to the Declaration of Helsinki and approved by the ethical committee of the Zhejiang Provincial Hospital of Traditional Chinese Medicine (TCM). Patients meeting the inclusion and exclusion criteria scheduled for CINV from July 2011 to September 2012 in Zhejiang Provincial Hospital of TCM were divided into two groups: sham-TEA (17 female, 17 male) and TEA group (12 females, 26 males). Written informed consent was obtained from all subjects before the study.

### 2.2. Inclusion and Exclusion Criteria

The inclusion criteria were as follows: (1) ages 18–80 years with confirmed diagnosis of cancer; (2) either being naive to chemotherapy or having received only moderately or highly emetogenic chemotherapy; (3) being scheduled to receive one cycle of moderately or highly emetogenic chemotherapy (≥50 mg/m^2^ cisplatin, >1500 mg/m^2^ cyclophosphamide, and >250 mg/m^2^ Carmustine); (3) Karnofsky's score ≥60; (4) white blood cell ≥3 × 10^9^/L and adequate hepatorenal function, aspartate aminotransferase <100 IU/l, alanine aminotransferase <100 IU/l, and creatinine clearance ≥60 mL/min; and (5) being scheduled to stay at hospital for chemotherapy.

Exclusion criteria included the following: (1) receiving concurrent radiotherapy of the upper abdomen or cranium; (2) vomiting or ≥grade 2 nausea (the National Cancer Institute—Common Terminology Criteria for Adverse Events v3.0 (CTCAE)) not clear to me; (3) severe uncontrolled complications; (4) unstable metastases in the brain; (5) uncontrolled pleural effusion or ascites; (6) gastrointestinal obstruction; (7) unwillingness or inability to accept acupuncture treatment, such as wrist disability or hematonosis; (8) contraindications to 5-HT_3_ receptor antagonists; (9) history of convulsions or seizure disorder; and (10) inability to understand or cooperate with study procedures.

### 2.3. Treatment Regimens

At the beginning of the study, patients who met all entry criteria were assigned to either TEA or sham-TGEA group according to a computer generated randomization schedule. The patients in the TEA group were treated with TEA at acupoints PC 6 and PC 5, whereas the patients in the sham-TEA group were treated with the same electrical stimulation at sham-points (neither on acupoints nor on any meridians). Sham-point 1 was at the lateral end of the transverse cubital crease, 2 cun (50 mm) from the bicipital muscle of arm; sham-point 2 was at medial end of the transverse cubital crease, condylus medialis humeri. The treatment was given twice daily each lasting one hr using a special watch-size stimulator (SNM-FDC01, Ningbo MaiDa Medical Device Inc., Ningbo, China) with the following parameters: monophasic, rectangular-wave pulses with pulse width of 0.3 ms, frequency of 20 Hz, and amplitude of up to 10 mA (individually adjusted according to the tolerance of the subject). The stimulation was delivered intermittently with on-time of 0.1 s and off-time of 0.4 ms. This set of parameters was previously used in animals to exert antiemetic [16] and analgesic effects [17]. Both groups received granisetron (3 mg iv bid) during the three-day treatment.

### 2.4. Clinical Efficacy

Nausea and vomiting were noted starting from administration of moderately or highly emetogenic chemotherapy up to 3 days. Patients recorded the date and time of episodes of emesis and the degree of nausea in diaries. The definition of an emetic episode was as follows: one episode of vomiting or a sequence of episodes in very close succession not relieved by a period of at least one min relaxation; any number of retching episodes in any given 5 min period; or an episode of retching lasting <5 min combined with vomiting not relieved by a period of relaxation of at least 1 min [18]. Nausea was classified into four grades (0: none; 1: mild; 2: moderate; and 3: severe). Any use of rescue medications was recorded, including drug name, dose, and time of administration. Rescue medication was administered for an emetic event or nausea upon request of the patient. The patients' diaries were checked daily by research staff for accuracy and completion.

Clinical efficacy was assessed as follows: (1) the proportion of patients with complete response (CR): no emesis and no rescue medications during the acute phase (0–24 h) after chemotherapy; (2) the proportion of patients with CR during the delayed phase (24–72 h) after chemotherapy; (3) the proportion of patients with complete control (CC): no emetic episode, no rescue medication, and no more than mild nausea during the delayed phase (24–72 h) after chemotherapy.

### 2.5. Mechanistic Measurements

Blood samples were collected at 6 AM on day 1 and day 3 after overnight fasting using tubes with EDTA and Aprotinin, centrifuged at 4200 g and 4°C for 10 min, and stored at 4°C until extraction. Plasma levels of 5-HT and dopamine were determined with the corresponding commercial ELISA kits (Beifang Institute of Biology and Technology, Beijing Rigorbio Science Development Co., Ltd., Beijing, China).

### 2.6. Safety Measurements

Vital signs (body temperature, heart rate, and respiratory rate), 12-lead electrocardiogram, blood tests (white blood cell, aspartate aminotransferase, alanine aminotransferase, and creatinine clearance), and urinalysis were assessed on days 1 and 3. Safety was also assessed by recording adverse events (AEs) up to 14 days after the therapy. AEs were assessed using common terminology criteria for adverse events (CTCAE) v4.0 by the investigators for intensity [19, 20].

### 2.7. Statistical Methods

All data are presented as mean ± SEM. Student's *t*-test was used to determine the difference between before and after the treatment in any measurement (nausea score, 5-HT, or dopamine level) and the difference in any measurement between the two treatments (SPSS 17.0 for Windows-standard version; SPSS Inc., Chicago, IL, USA). Fisher's exact test was used to compare the clinical efficacy of the two treatment methods (TEA versus sham-TEA). Statistical significance was assigned for *P* < 0.05.

## 3. Results

### 3.1. Effects on Nausea and Vomiting

**Figure 1:**
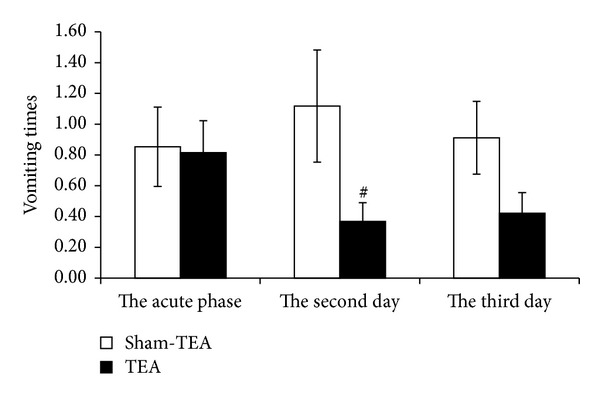
Effect of TEA on vomiting times. TEA significantly reduced the vomiting times on the second day after chemotherapy compared to sham-TEA group and reduced it on the third day after chemotherapy, but the difference was not significant (^#^ *P* < 0.05).

TEA improved vomiting in the delayed phase although it did not in the acute phase. The average number of vomiting episodes was 0.85 ± 0.26 with sham-TEA and 0.82 ± 0.20 with TEA (*P* = 0.9) in the first 24 hours (acute phase) (*P* = 0.9). In the delayed phase, however, this number was significantly lower with TEA than sham-TEA (*P* = 0.046 for the second day and *P* = 0.68 for the third day) (see Figure 1).

**Figure 2:**
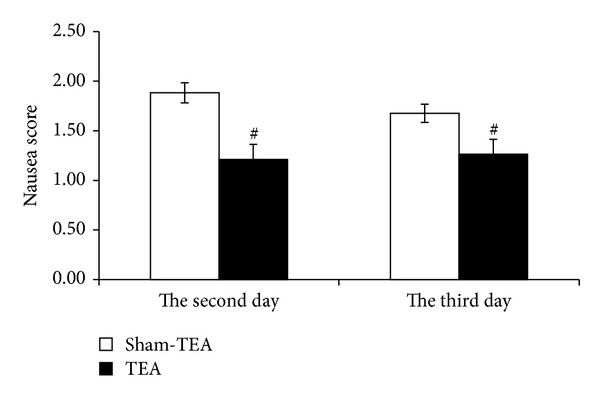
TEA reduced the nausea scores at both 48 h and 72 h after chemotherapy. TEA reduced substantially the nausea scores by 55.5% at 48 h and significantly by 32.7% at 72 h compared to sham-TEA group (^#^ *P* < 0.05).

The nausea scores during the delayed phase (48 h, 72 h) were 1.88 ± 0.10 and 1.68 ± 0.10 in the sham-TEA group and 1.21 ± 0.15 and 1.26 ± 0.15 in the TEA group, respectively (Figure 2). The differences between two groups were significant (*P* = 0.001 and 0.025, resp.). No significant difference was noted in the rate of complete response between the two groups, neither in the acute phase nor in the delayed phase.

The rate of complete control was significantly increased with TEA during the second day as shown in Table 1 (*P* = 0.008 for the second day and *P* = 0.3 during the third day).

### 3.2. Mechanisms Involving Serotonin and Dopamine

**Figure 3:**
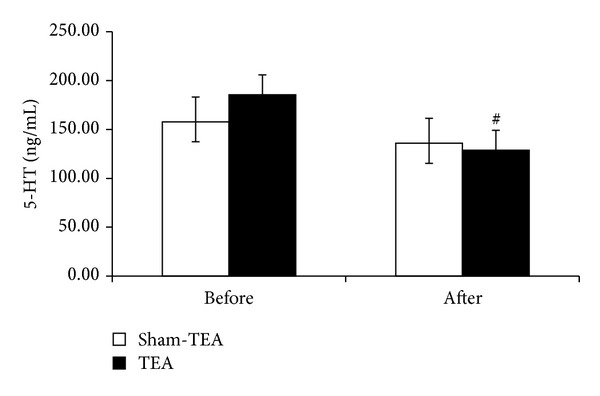
Effect of TEA on serum levels of 5-HT before and after the treatment. TEA significantly reduced the serum level of 5-HT compared to sham-TEA (^#^ *P* < 0.05).

**Figure 4:**
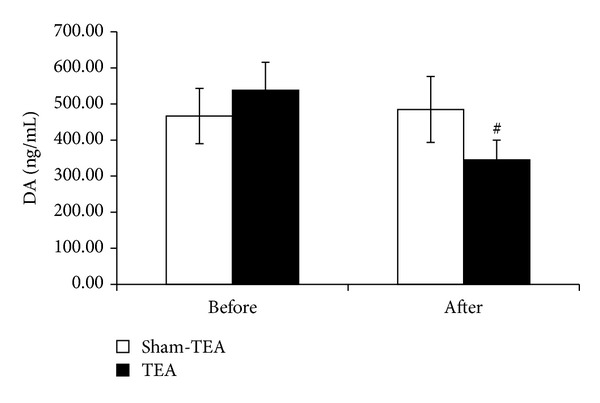
Effect of TEA on serum levels of DA before and after the treatment. There are significant differences of serum level of DA between TEA and sham-TEA (^#^ *P* < 0.05).

TEA significantly reduced circulating 5-HT and dopamine. At baseline, no difference was noted in serum 5-HT and dopamine levels between the TEA and sham-TEA groups. After the treatment, however, the serum levels of 5-HT and dopamine were significantly reduced (*P* = 0.03 and *P* = 0.02, resp.) (Figures 3 and 4).

### 3.3. Adverse Events

Safety was assessed in all patients. Laboratory examinations (white blood cell, aspartate aminotransferase, alanine aminotransferase, and creatinine clearance) and electrocardiogram were found normal after the treatment in all patients (both groups) except one who had allergic reaction of medical adhesive tape judged to be unrelated or unlikely related to TEA.

## 4. Discussion

In this study, we found that TEA at PC6 and PC5 reduced nausea and vomiting in the delayed phase of chemotherapy in patients with cancers. This antiemetic effect was possibly mediated via mechanisms involving serotonin and dopamine.

Various methods of acupuncture have been applied for treating CINV, such as manual acupuncture, acupressure, electroacupuncture, auricular acupuncture, and pharmacopuncture. Dundee et al. were the first ones who reported the antiemetic effect of acupuncture on CINV [14, 15]. Recently it was reported that acupressure also exerted an antiemetic effect on CINV in patients with breast cancers [21]. Auricular acupuncture was applied to treat CINV in children with cancers who underwent chemotherapy and shown to be effective but not different from sham stimulation [22]. A recent review on pharmacopuncture (medications delivered via the acupoints) analyzed 22 studies involving about 2500 patients but failed to provide a confirmative conclusion due to high risk of bias and clinical heterogeneity [23]. Although acupuncture and its variations are promising in treating CINV, no definitive conclusions could be made from studies reported in the literature due to poor study design and high risk of bias. In a recent systematic review of acupuncture in cancer care, a total of 2,151 publications were screened; it was concluded that acupuncture was an adequate complementary therapy for CINV but additional studies were needed [24].

In this study, a needleless method of TEA was introduced and a placebo controlled clinical trial was designed to investigate the antiemetic effect of TEA on CINV in patients with cancers. A special set of parameters was used based on a previous study in our lab with gastric electrical stimulation showing an antiemetic effect in dogs treated with cisplatin and an analgesic effect in rats with gastric hypersensitivity [16, 17]. Using these special settings we found that TEA was able to significantly improve delayed emesis and nausea during the second day of the treatment. No significant effect was noted in the acute phase, attributed to the fact that Ondansetron effectively controlled emesis during the first day of the chemotherapy. Previously, acupuncture and electroacupuncture were shown to improve gastric motility and symptoms of upper abdomen, such as nausea and vomiting. In canine study we found that electroacupuncture at PC6 reduced vasopressin-induced nausea and vomiting mediated via the vagal mechanism [25]. Ouyang et al. reported that electroacupuncture at points PC6 and ST36 significantly accelerated gastric emptying in dogs also mediated via the vagal mechanism [26]. Clinically, there is evidence that acupuncture at PC6 and ST36 improved dyspeptic symptoms including nausea and vomiting and accelerates solid gastric emptying in patients [27]. These findings seem to suggest that electroacupuncture or TEA is capable of improving nausea and vomiting of different causes.

To the best of our knowledge, this was the first study investigating and demonstrating the antiemetic mechanisms of TEA involving 5-HT and dopamine. Serotonin and dopamine are two main neurotransmitters known to induce CINV. Many drugs of chemotherapy can cause emesis and nausea via upregulation of 5-HT and dopamine, and antagonists of serotonin and dopamine are commonly used in CINV [28, 29], and antagonists of serotonin are more common than antagonists of dopamine in treatment of CINV. Ondansetron, a 5-HT_3_ antagonist, was used in this study as the primary antiemetic. It effectively reduced the number of vomiting times to an average level of 1. Interestingly, TEA was found to reduce circulating 5-HT in comparison with sham-TEA. Exact mechanisms involved in the reduction of 5-HT with TEA deserve further investigation. In gastrointestinal motility study, electroacupuncture was found to accelerate gastric emptying mediated via the 5-HT mechanism [18]. It was reported that electroacupuncture on the lumbar and hindlimb segments decreased the dopamine and serotonin levels which were increased by restraining stress in the dorsal raphe nucleus, indicating that electroacupuncture applied to the lumbar and hindlimb segments has an antistress effect via mediation of the levels of serotonin and dopamine [30]. However, different subtypes of 5-HT receptors are believed to be involved in the antiemetic effect and the prokinetic effect of acupuncture. The prokinetic effect of acupuncture is believed to involve 5-HT_4_ mechanism, whereas the antiemetic effect of acupuncture is believed to involve 5-HT_3_ mechanisms [29, 31]. In addition, a reduction in circulating dopamine was also noted after the treatment of TEA. This reduction might also play a role in the antiemetic effect of TEA. The mechanism involving dopamine was reported in the effect of acupuncture on drug addiction [32]; it was, however, first reported in this study regarding the effect of acupuncture on CINV.

Traditional acupuncture or electroacupuncture treatment needs to be done in clinics and needle should be pierced into points. In this study, TEA did not require the insertion of any needles and the patient's activity was not restricted. So TEA seems to be more attractive than acupuncture or electroacupuncture and will be well received by patients. In this study, the compliance of the therapy was 100%; none of the patients quitted the study. Typically, acupuncture or electroacupuncture is performed a few times weekly due to required visits to doctor's office. This substantially reduces the efficacy and consistency of the therapy. With the TEA method, the treatment can be self-administrated at home and thus could be performed daily or a few times daily, which would greatly increase the efficacy of the therapy.

## 5. Conclusions

In conclusion, a needleless method of transcutaneous electroacupuncture is proposed in this study. The needleless TEA is effective in reducing delayed nausea and vomiting in patients undergoing chemotherapy, possibly mediated via the downregulation of serotonin and dopamine.

## Figures and Tables

**Table 1:** Patients with the CC rates in delayed emesis (48 h; 72 h; case%).

The second day		The third day	
Sham-TEA	TEA	Sham-TEA	TEA
8 (23.6%)	21 (55.3%)^#^	12 (35.3%)	18 (47.4%)

The rate of complete control was significantly increased with TEA during the second day compared to sham-TEA (^#^ *P* < 0.01).
